# Spectrum and clinical features of gene mutations in Chinese pediatric acute lymphoblastic leukemia

**DOI:** 10.1186/s12887-023-03856-y

**Published:** 2023-02-04

**Authors:** Diying Shen, Lixia Liu, Xiaojun Xu, Hua Song, Jingying Zhang, Weiqun Xu, Fenying Zhao, Juan Liang, Chan Liao, Yan Wang, Tian Xia, Chengcheng Wang, Feng Lou, Shanbo Cao, Jiayue Qin, Yongmin Tang

**Affiliations:** 1 Pediatric Hematology-Oncology Center, Zhejiang Provincial Center for Childhood Leukemia Diagnosis and Treatment, The Children’s Hospital, Zhejiang University School of Medicine, National Clinical Research Center for Child Health, Hangzhou, China; 2 Acornmed Biotechnology Co., Ltd, Tianjin, China

**Keywords:** Acute lymphoblastic leukemia, Gene mutations, Clinical features, Correlations, Next-generation sequencing

## Abstract

**Purpose:**

The 5-year survival rate of children with acute lymphoblastic leukemia (ALL) is 85–90%, with a 10–15% rate of treatment failure. Next-generation sequencing (NGS) identified recurrent mutated genes in ALL that might alter the diagnosis, classification, prognostic stratification, treatment, and response to ALL. Few studies on gene mutations in Chinese pediatric ALL have been identified. Thus, an in-depth understanding of the biological characteristics of these patients is essential. The present study aimed to characterize the spectrum and clinical features of recurrent driver gene mutations in a single-center cohort of Chinese pediatric ALL.

**Methods:**

We enrolled 219 patients with pediatric ALL in our single center. Targeted sequencing based on NGS was used to detect gene mutations in patients. The correlation was analyzed between gene mutation and clinical features, including patient characteristics, cytogenetics, genetic subtypes, risk stratification and treatment outcomes using χ^2^-square test or Fisher’s exact test for categorical variables.

**Results:**

A total of 381 gene mutations were identified in 66 different genes in 152/219 patients. *PIK3R1* mutation was more common in infants (*P* = 0.021). *KRAS* and *FLT3* mutations were both more enriched in patients with hyperdiploidy (both *P* < 0.001). *NRAS*, *PTPN11*, *FLT3*, and *KMT2D* mutations were more common in patients who did not carry the fusion genes (all *P* < 0.050). *PTEN* mutation was significantly associated with high-risk ALL patients (*P* = 0.011), while *NOTCH1* mutation was common in middle-risk ALL patients (*P* = 0.039). Patients with *ETV6* or *PHF6* mutations were less sensitive to steroid treatment (*P* = 0.033, *P* = 0.048, respectively).

**Conclusion:**

This study depicted the specific genomic landscape of Chinese pediatric ALL and revealed the relevance between mutational spectrum and clinical features of Chinese pediatric ALL, which highlights the need for molecular classification, risk stratification, and prognosis evaluation.

**Supplementary Information:**

The online version contains supplementary material available at 10.1186/s12887-023-03856-y.

## Introduction

Pediatric acute lymphoblastic leukemia (ALL) is the most common childhood malignancy with 5-year overall survival (OS) rate of 85–90% and treatment failure rate of 10–15% [1–4]. Next-generation sequencing (NGS) identified recurrent mutated genes in pediatric ALL that might alter the diagnosis, classification, prognostic stratification, treatment, and response to ALL. However, there are few studies on gene mutations in Chinese pediatric ALL. Thus, an in-depth understanding of the biological characteristics of these patients is essential, and it is necessary to conduct comprehensive and thorough gene mutation detection by NGS in ALL patients. In the current study, we aimed to characterize the spectrum and clinical features of gene pathogenic variants in a single-center cohort of Chinese pediatric ALL patients.

## Methods

### Patients

The cohort in the present study consisted of 219 consecutive children (0.05–16.25, median: 3.75 years) with newly diagnosed ALL (*n* = 196, B-cell ALL (B-ALL); *n* = 23, T-cell ALL (T-ALL)) receiving treatment at our hospital between October 2017 and October 2019 and for whom complete data were available. The protocol was approved by the Medical Ethics Committee of the Children’s Hospital, and written informed consent was obtained from the parents or guardians. ALL was diagnosed based on the morphology, immunophenotyping, cytogenetics, and the molecular biology of leukemia cells [5]. Flow cytometric (FCM) immunophenotyping of bone marrow was performed on FACSCalibur with CellQuest software. A total of 51 fusion genes, including *ETV6-RUNX1*, *BCR-ABL1*, and *MLL* rearrangement, were examined by polymerase chain reaction (PCR). Karyotyping analysis was conducted by conventional methods. The patients were treated according to the National Protocol of Childhood Leukemia in China (NPCLC)-ALL2008 protocol, a modified form of protocol NPCAC97 [6].

### Risk stratification

In this study, patients were classified into three groups: low risk (LR), intermediate risk (IR), and high risk (HR). If any one of the following criteria were fulfilled, the patients were assigned to the HR group: (i) Patients < 1-year-old; (ii) *MLL* rearrangement or t (17; 19) [*TCF3/HLF*] or hypodiploidy (< 45 chromosomes); (iii) Altered *IKZF1*; (iv) Poor response to prednisone or did not reach complete remission (CR) at the end of induction therapy; (v) Minimal residual disease (MRD) ≥ 1% on day 15 or 33 of induction. For IR group, any of the following criteria need to be met: (i) Patients > 10-year-old; (ii) White blood cell (WBC) count > 50 × 10^9^ /L; (iii) T-ALL; (iv) *TCF3/PBX1*; (v) *Ph*^+^ ALL; (vi) *Ph-like* ALL; (vii) Central nervous system (CNS) 2 status (< 5 leukocytes/μL with blast cells in a cerebrospinal fluid sample with < 10 erythrocytes/μL), CNS3 status (≥ 5 leukocytes/μL with blast cells in a cerebrospinal fluid sample with < 10 erythrocytes/μL or the presence of a cerebral mass or cranial palsy) [7]; (viii) Testicular leukemia at diagnosis; (ix) 0.1% ≤ MRD ≤ 1% on day 15 of induction or 0.01% ≤ MRD ≤ 1% on day 33 of induction. The remaining patients with MRD < 0.01% on day 15 and 33 were categorized into the LR group.

### Minimal residual disease monitoring

MRD was determined by FCM (FACSCalibur), as described previously [8]. The antibodies used for staining were conjugated with any of the following fluorochromes: fluorescein isothiocyanate (FITC), phycoerythrin (PE), peridinin chlorophyll protein (PerCP), and allophycocyanin (APC). The appropriate combination of antibodies was selected for MRD detection, such as leukemia-associated immunophenotype (LAIP). For B-ALL, CD19/CD10/CD34/CD45 was the most common combination; meanwhile, CD19/CD10/CD45/CD20, CD19/CD20/CD34/CD45, and CD19/CD10/CD34/CD20 were also utilized. For T-ALL, CD34, CD7, CD3, terminal deoxynucleotide transferase (TdT), and HLA-DR were the primary markers, and the most frequently used antibody combinations were CD45/CD2/CD3/CD56 and CD45/CD7/CD3/CD56. The bone marrow MRD was detected on days 15 and 33 after the start of induction.

### Next-generation sequencing and mutation analysis

Genomic DNA was extracted from bone marrow samples at diagnosis. The spectrum of gene mutations was determined through NGS platform at Acornmed Biotechnology Co. Ltd. (Beijing, China). Genetic profiling included the targeted sequencing of 185 genes. Multiplex libraries were sequenced using Illumina NovaSeq. The following criteria were used to filter raw variant results: average effective sequencing depth on target per sample ≥ 1000x; variant allele frequency (VAF) ≥1% for single nucleotide variations (SNVs), insertions, or deletions (InDels); mapping quality ≥30; and base quality ≥30. The reads were aligned to the human genome using Burrows-Wheeler alignment (BWA, version 0.7.12). PCR duplicates were removed using the MarkDuplicates tool in Picard. Genome Analysis Toolkit (GATK; version 3.8) comprising of IndelRealigner and BaseRecalibrator was applied for realignment and recalibration of the BWA data, respectively. Mutect2 was used to identify SNVs and InDels. All the variants were annotated by ANNOVAR software, including 1000G projects, COSMIC, SIFT, and PolyPhen.

### Statistics

The correlations between various gene mutations and clinical features were analyzed using χ^2^-square test or Fisher’s exact test for categorical variables A two-sided *P* value < 0.05 was considered to indicate statistical significance.

## Results

### Spectrum of gene mutations in pediatric ALL patients

**Fig. 1:**
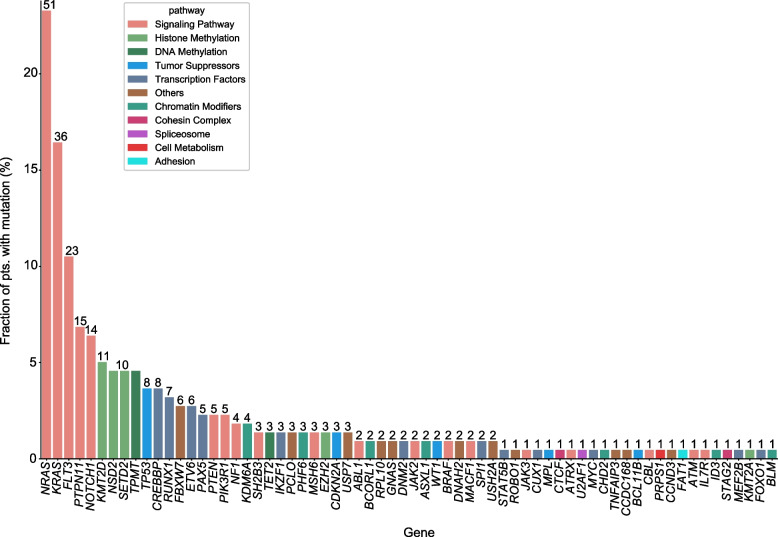
The landscape of gene mutations identified by next-generation sequencing (NGS) in 219 Chinese pediatric patients with acute lymphoblastic leukemia (ALL)

**Fig. 2:**
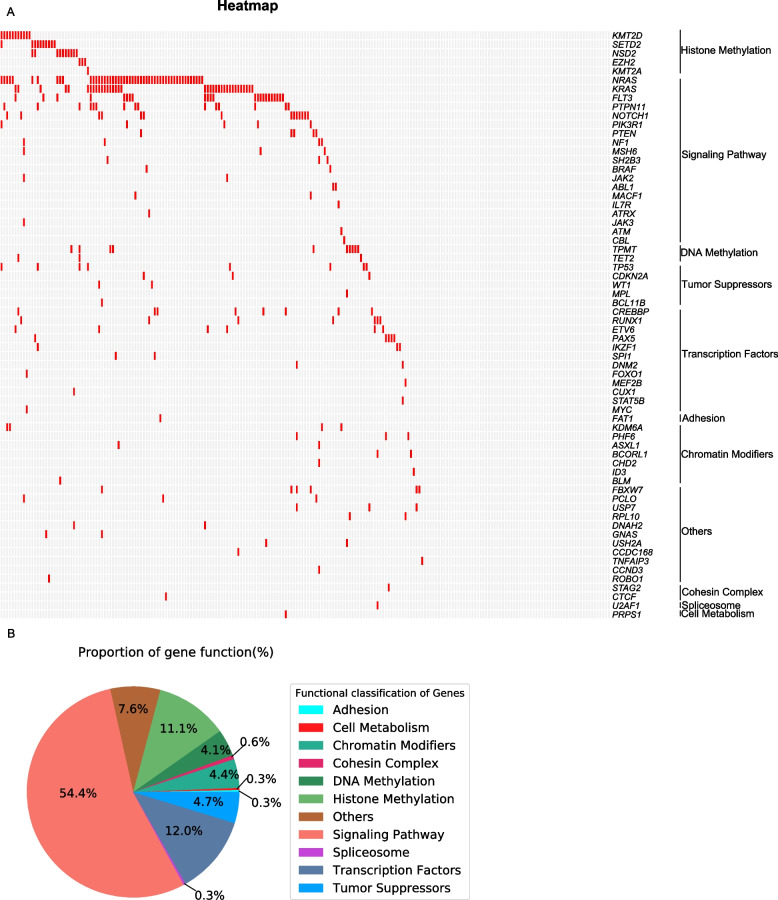
Functional pathways of mutated genes. **a** Heatmap representing mutated genes classified into different functional pathways. **b** Proportion of gene function groups

The baseline characteristics of the patients are listed Table 1. A total of 381 gene mutations, including SNVs and InDels, were identified in 66 different genes in 152/219 patients (69%) (Fig. 1). The average number of gene mutations was 2.5 (range 0–10)/patient, and the average number of mutations in T-ALL and B-ALL patients was 3 (0–10) and 1 (0–7), respectively. The mutated genes were comprised of several functional groups, mainly including 54.4% in signaling pathway, 12% in transcription factors, and 11% in DNA methylation (Fig. 2). The mutated genes with the gene mutation fraction of patients ≥5% in our cohort were *NRAS* (*n* = 51, 23%), *KRAS* (*n* = 36, 16%), *FLT3* (*n* = 23, 11%), *PTPN11* (*n* = 15, 7%), *NOTCH1* (*n* = 14, 6%) and *KMT2D* (*n* = 11, 5%), respectively (Fig. 1).

**Table 1:** Clinical features

Variable	Total cohort, ***n*** = 219
Gender, n (%)	
Male	121 (55%)
Female	98 (45%)
Age (year, median, range)	3.75 (0.05–16.25)
Infant, n (%)	11 (5%)
≥ 1, n (%)	208 (95%)
WBC (× 10^9^/L, median, range)	7.85 (0.35–912.25)
HB (×g/L, median, range)	82 (27–159)
PLT (×10^9^/L, median, range)	62 (3–483)
Immunophenotype, n (%)	
B-ALL	196 (89%)
T-ALL	23 (11%)
Risk stratification defined group, *n* = 217, n (%)	
Low risk	76 (35%)
Intermediate risk	84 (38%)
High risk	57 (26%)
Cytogenetics, n (%)	
Hyperdiploid	62 (28%)
Genetic fusion subtype, n (%)	
*ETV6*-*RUNX1*	47 (20.4%)
*BCR*-*ABL1*	7 (3.1%)
*TCF3*-*PBX1*	10 (4.5%)
*MLL* rearrangement	11 (5%)

Abbreviations: WBC, white blood cell; Hb, hemoglobin; PLT, platelet; B-ALL, B-cell acute lymphoblastic leukemia; T-ALL, T-cell acute lymphoblastic leukemia

**Fig. 3:**
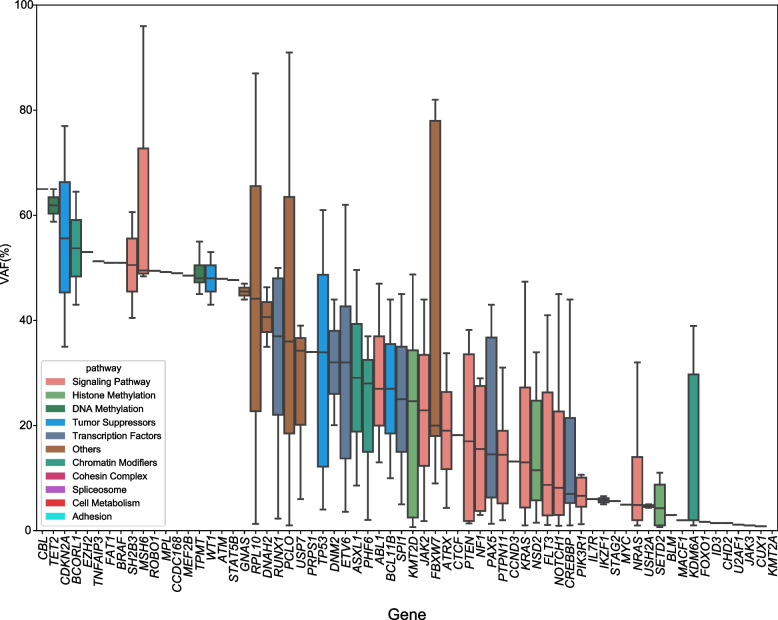
Variant allele frequency (VAF) analysis of various gene mutations

The median VAF of gene mutations in the 219 ALL patients was 20% (Fig. 3). The mutated genes with median VAF ≥ 25% included *CBL*, *TET2*, *CDKM2A*, *BCORL1*, *EZH2*, *TNFAIP3*, *FAT1*, *BRAF*, etc. Conversely, the rest of the mutated genes with lower VAF < 25% indicated that they were present in a subpopulation of the sequenced cells. Notably, four RAS signaling pathway mutated genes (*FLT3*, *NRAS*, *KRAS*, and *PTPN11*) had lower VAFs < 25%, suggesting that these genes were subclones. The mutations mainly affected codons 12 and 13 of *KRAS* and *NRAS*. The allele frequency of different hotspot mutations was compared, and it was found that the frequency of *KRAS G12D* was higher than that of other hotspot mutations (Supplemental Fig. S1), which was similar to the results reported in some previous literatures [9].

**Fig. 4:**
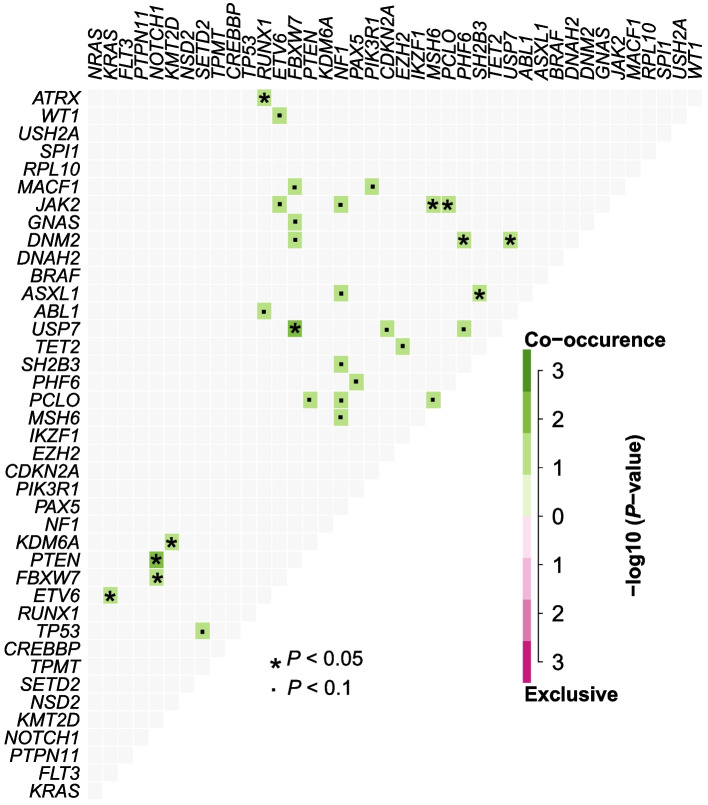
Correlation analysis of pairwise co-occurring mutated genes

Next, we investigated the co-occurrence of mutated genes and found significant associations between mutated *NOTCH1* and mutations in *FBXW7* and *PTEN*, mutated *JAK2* and mutations in *MSH6* and *PCLO,* and mutated *DNM2* and mutations in *PHF6* and *USP7*. Moreover, pairwise associations were observed between *ETV6* and *KRAS*, *KDM6A* and *KMT2D*, *RUNX1* and *ATRX*, *ASXL1* and *SH2B3,* and *USP7* and *FBXW7* (*P* < 0.05, Fig. 4)*.*

### Correlation between gene mutations and patient characteristics, cytogenetics

**Fig. 5:**
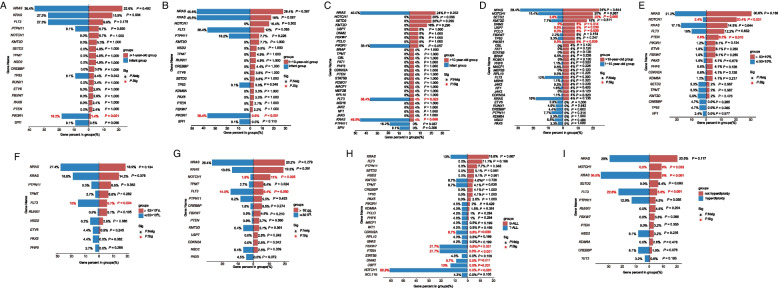
Correlation between gene mutations and patient characteristics and cytogenetics. **a**-**d** Association of gene mutations with different age groups of patients. (E-G) Association of gene mutations with white blood cell **e**, platelet **f**, and hemoglobin **g** groups, respectively. **h** Different distribution of gene mutations in T-ALL and B-ALL. **i** Association of gene mutations between hyperdiploidy and not hyperdiploidy groups

The correlation analysis between gene mutations and the patient characteristics in these patients revealed that *PIK3R1* mutation was more common in infants compared to patients ≥1-year-old (*P* = 0.021, Fig. 5A). Moreover, *PIK3R1*was more common in infants, compared with 1 ~ 10-year-old group (*P* < 0.001, Fig. 5B).While compared with infant group, both *FLT3* and *KRAS* mutations were less common in patients > 10-year-old (*P* = 0.023, *P* = 0.006, respectively, Fig. 5C). At the same time, we discovered *PIK3R1, PCLO, USP7, DNM2 and SETD2* mutations were more common in > 10-year-old group, compared with 1 ~ 10-year-old group (all *P* < 0.05, Fig. 5D). We also found that gender did not influence the mutational status of any of the genes. In addition, the mutations in *NOTCH1* and *PTEN* were more common in patients with initial leukocyte count > 50 × 10^9^/L (*P* < 0.001 and *P* = 0.097, respectively) (Fig. 5E). Patients with *FLT3* mutations showed lower platelet counts (≤62 × 10^9^/L, *P* = 0.024, Fig. 5F) and hemoglobin level (≤82 g/L, *P* = 0.049, Fig. 5G) at diagnosis than those without *FLT3* mutations. While patients with *NOTCH1* mutations had a high hemoglobin level (> 82 g/L, *P* = 0.005) (Fig. 5E). Compared to B-ALL, *NOTCH1*, *PTEN*, *FBXW7*, *USP7*, *DNM2*, and *CDKN2A* were more frequently mutated in T-ALL (all *P* < 0.05) (Fig. 5H). Futhermore, *KRAS* and *FLT3* mutations were both enriched in patients with hyperdiploidy (both *P* < 0.001, Fig. 5I).

### Correlation between gene mutations and genetic subtypes, risk stratification and treatment outcomes

**Fig. 6:**
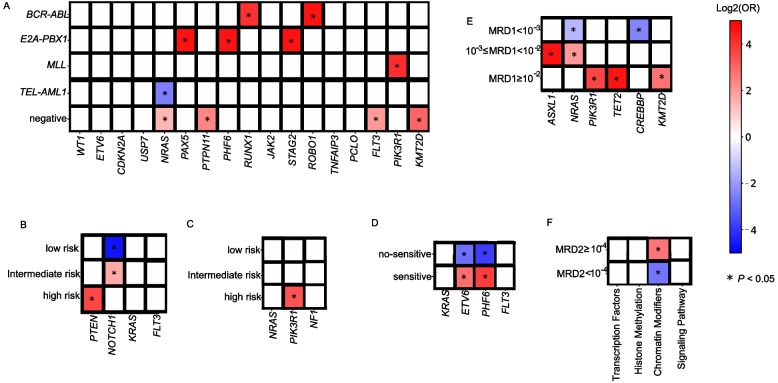
Correlation between gene mutations and genetic subtypes, risk stratification and treatment outcomes. **a** Association of gene mutations with genetic fusion subtypes. **b** Association of gene mutations with risk stratification in all ALL patients. **c** Association of gene mutations with risk stratification in B-ALL. **d** Association of gene mutations with steroid therapeutic effects. **e** Association of gene mutations with MRD level in B-ALL. **f** Association between gene groups and MRD2 in B-ALL

Molecular genetic analyses of 51 fusion transcripts, including *ETV6*-*RUNX1*, *TCF3*-*PBX1*, *BCR*-*ABL1*, and *KMT2A* (*MLL*) rearrangement were conducted successfully in all the patients. Strikingly, *NRAS*, *PTPN11*, *FLT3*, and *KMT2D* mutations were common in patients who did not carry the fusion genes (all *P* < 0.050), and *NRAS* mutations were rarely in patients with *ETV6*-*RUNX1* (*P* = 0.002). *RUNX1* and *ROBO1* mutations were more closely linked to *BCR-ABL1* fusion gene (*P* = 0.017, *P* = 0.032, respectively), and *PAX5*, *PHF6*, and *STAG2* mutations were associated with *TCF3*-*PBX1* fusion gene (*P* = 0.001, *P* = 0.006, *P* = 0.046, respectively). *MLL* translocations co-existed with *PIK3R1* mutation (*P* = 0.017, Fig. 6A). Some genes are closely associated with the prognosis of the disease. Herein, we studied the associations of gene mutations with risk stratification in the cohort. *PTEN* mutation was significantly associated with high risk ALL patients (*P* = 0.011), while *NOTCH1* mutation was common in intermediate risk ALL patients (*P* = 0.039, Fig. 6B). In addition, *PIK3R1* mutation occurs frequently in high risk B-ALL patients (*P* = 0.023, Fig. 6C). Patients with *ETV6* or *PHF6* mutations detected at the time of diagnosis were less sensitive to steroid treatment (*P* = 0.033, *P* = 0.048, respectively, Fig. 6D). In B-ALL patients, we analyzed the associations between gene mutations and early MRD levels (MRD1 on day 15 and MRD2 on day 33). Patients with mutated *PIK3R1*, *TET2*, and *KMT2D* had a markedly higher MRD1 level (MRD1 ≥ 10^− 2^). *ASXL1* and *NRAS* mutations were enriched in patients with 10^− 3^ ≤ MRD1 < 10^− 2^ (*P* = 0.024, *P* < 0.001, respectively), whereas *NRAS* and *CREBBP* mutations were less significantly in B-ALL patients with MRD1 < 10^− 3^ (*P* = 0.005, *P* = 0.042, Fig. 6E). However, no significant association was established between gene mutations and MRD2, but the patients carrying the genes with mutated chromatin modifiers exhibited a significantly high level of MRD2 (MRD ≥10^− 4^, 21.4% vs. 4%, *P* = 0.029, Fig. 6F).

## Discussion

In this study, we dissected the genetic landscape, analyzed the mutational spectrum of various immunological ALL lineages, and explored the correlations between mutational and clinical features, including patient characteristics, risk stratification, and treatment outcomes in a Chinese pediatric ALL cohort. A number of gene pathogenic variants were identified, which provided a comprehensive genomic profile of Chinese pediatric ALL. Consistent with previous reports, B-ALL and T-ALL presented a distinct mutation spectrum; Ras pathway mutations were enriched in B-ALL, while Notch pathway mutations were enriched in T-ALL [10–13].

As described previously, mutations involved in the *Ras* signaling pathway (*NRAS*, *KRAS*, *FLT3*, *PTPN11*, and *NF1*) occurred in more than half of B-ALL patients [14, 15] . Also, a higher incidence of mutations was detected in *NRAS* rather than *KRAS*. This finding was contradictory to the previous studies in the Chinese cohort [15] but was in agreement to that from the USA, Sweden, and Korea [14–19]. These discrepancies might be related to the population distribution and environmental factors, which highlighted the genetic heterogeneity of pediatric ALL. Compared to *CBL*, *TET2*, *CDKM2A*, and *BCORL1* genes with a higher median VAF, Ras signaling pathway-related genes, such as *FLT3*, *NRAS*, *KRAS*, and *PTPN11*, displayed a lower median VAF of 5–20%. The lower VAF indicated that Ras mutations were more likely subclones rather than a major clone [20], suggesting that B-ALL is driven by other fusion genes. Reportedly, *Ras* pathway functioned as a molecular switch for signaling pathways that regulated cell proliferation, survival, growth, migration, and differentiation [21]. Thus, we speculated that *Ras* pathway mutations occurred during B-ALL progression rather than tumorigenesis. Based on genetic testing of a large number of ALL patients, Shu et al. and Perentesis et al. demonstrated that *RAS* mutations did not present any unique clinical manifestation nor predicted clinical outcomes [18, 19]. Moreover, some recent studies showed that ALL patients with *Ras* pathway mutations, especially *KRAS*/*NRAS* mutations, present high-risk features, including early relapse and CNS involvement [22–24]. In our cohort, no correlation was established between the presence of Ras mutation and clinical characteristics, risk stratification, and MRD level. This phenomenon could be attributed to the neutralization effect of other genomic variations, such as low-risk hyperdiploidy and high-risk hypodiploidy on prognosis [25–28]. To determine whether Ras pathway status influences the clinical characteristics and risk stratification, additional studies are warranted on various cytogenetic subgroups of B-ALL. Notch pathway mutations, especially *NOTCH1* and *FBXW7*, were enriched in T-ALL patients, as previously reported [29–32]. *NOTCH1* was the most common mutated gene in about 60.9% of all T-ALL cases, followed by *PTEN* (21.7%) and *FBXW7* (21.7%). Notch signaling pathway, especially *NOTCH1*, plays a crucial role in all stages of T lymphocyte development and can promote the differentiation of lymphoid precursor cells into T lymphocytes and inhibit their differentiation into B lymphocytes [33, 34]. Except for the excessive activation of the Notch pathway, impaired *CDKN2A/2B* cell cycle regulators also played a prominent role in T-ALL pathogenesis. Strikingly, *CDKN2A*/*2B* deletions were detected in > 50% of T-ALL cases [10, 35]. However, the copy number variations were not detected and analyzed in the present study, and only a few *CDKN2A* gene point mutations were identified in T-ALL. Recent sequencing studies demonstrated that T-ALL was an aggressive malignancy caused by the accumulation of genomic lesions. On average, 10–20 mutations were detected in T-ALL cells [10, 36–38]. Although our study showed that T-ALL had a significantly higher mutation level than B-ALL, the average number of mutations was still lower than the expected value. This deviation could be attributed to the scope of sequencing, the evaluated variation types, the sensitivity of the test, and the filter criteria of mutation calling. We also found that the accumulation of mutations in T-ALL did not occur randomly [39]. Interestingly, the coexistence of *NOTCH1-PTEN-FBXW7* and *DNM2-USP7-PHF6* mutations was observed in our T-ALL cohort. The coexistence phenomenon suggested that those Notch pathway and non-Notch pathway genes interconnect physiologically and cooperate during the development and progression of the T-ALL, respectively. *MLL* translocations and *PIK3R1* mutations were common in infant ALL, a group characterized as immature cytologically, resistant to conventional therapies, and showing poor prognosis. Moreover, a significant coexistence between *MLL* gene arrangement and *PIK3R1* mutations was detected in our cohort. This observation indicated that *PI3K/AKT* is a secondary hit for partial *MLL*-positive ALL.

In this study, we wanted to analyze the relationship between these pathogenic variants and prognosis. Due to the short follow-up time, we only analyzed the relationship between MRD of day 15, 33 of induction and mutations. MRD is showed a high level in patients with *PIK3R1*, *TET2*, and *KMT2D* mutations, indicating a high risk of relapse. Both *TET2* and *KMT2D* belong to epigenetic regulator genes, which play key roles in DNA demethylation and histone H3 methylation, respectively [13, 40]. This finding suggested that mutations in epigenetic regulator genes elevate the MRD level. Future studies we will continue to explore the relationships between long-term prognosis, recurrence rates and pathogenic variants, which may help improve the prognosis of ALL in children.

In summary, our study depicted the specific genomic landscape and revealed the relevance between mutational spectrum and clinical features of Chinese pediatric ALL in a single cohort, including patient characteristics, cytogenetics, genetic subtypes, risk stratification and treatment outcomes. The discovery of this mutational spectrum highlights the need for molecular classification, risk stratification, and prognosis evaluation and also provide the basis for the development and application of new targeted therapy for pediatric ALL.

## Supplementary Information

**Additional file 1:** **Figure S1.** The variant allele frequency (VAF) of different KRAS and NRAS hotspot mutations.

**Additional file 2:** **Table S2.**

## Data Availability

Data and materials are available in supplementary file 1.

## Abbreviations

NGS: Next-generation sequencing
ALL: acute lymphoblastic leukemia
OS: overall survival
FCM: flow cytometric
PCR: polymerase chain reaction
LR: low risk
IR: intermediate risk
HR: high risk
CR: complete remission
MRD: minimal residual disease
WBC: white blood cell
CNS: central nervous system
FITC: fluorescein isothiocyanate
PE: phycoerythrin
PerCP: peridinin chlorophyll protein
APC: allophycocyanin
LAIP: leukemia-associated immunophenotype
VAF: variant allele frequency
SNVs: single nucleotide variations
BWA: Burrows-Wheeler alignment
GATK: Genome Analysis Toolkit
InDels: deletions
B-ALL: B-cell ALL
T-ALL: T-cell ALL
TdT: terminal deoxynucleotide transferase
