# Supplementary figures and images for: Spectrum and clinical features of gene mutations in Chinese pediatric acute lymphoblastic leukemia
Source: BMC Pediatr. 2023 Feb 4;23:62. doi: 10.1186/s12887-023-03856-y (PMC9898934; doi:10.1186/s12887-023-03856-y)

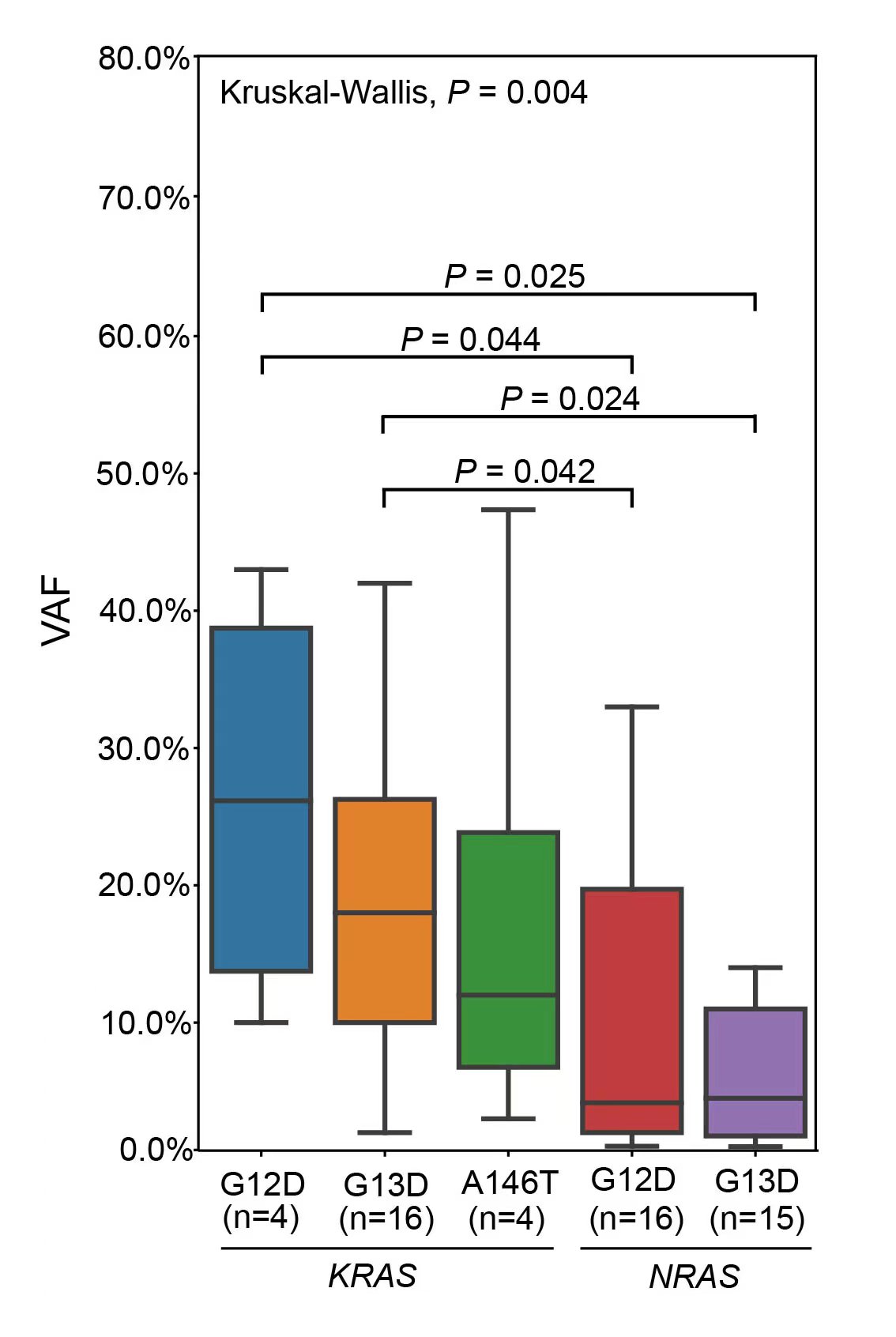


Figure S1 The variant allele frequency (VAF) of different KRAS and NRAS hotspot mutations.

Supplement: Supplementary file 1 — Additional file 1: Figure S1. The variant allele frequency (VAF) of different KRAS and NRAS hotspot mutations. [file 12887_2023_3856_MOESM1_ESM.docx]
